# 10.2 REDOX DYSREGULATION, OLIGODENDROCYTES AND WHITE MATTER ALTERATIONS IN SCHIZOPHRENIA

**DOI:** 10.1093/schbul/sby014.035

**Published:** 2018-04-01

**Authors:** Paul Klauser, Philipp S Baumann, Margot Fournier, Lijing Xin, Alessandra Griffa, Martine Cleusix, Raoul Jenni, Michel Cuenod, Patric Hagmann, Philippe Conus, Kim Q Do

**Affiliations:** 1 Lausanne University Hospital; 2 Center for Psychiatric Neuroscience, Lausanne University Hospital; 3 Animal Imaging and Technology Core (AIT), Center for Biomedical Imaging (CIBM), Ecole Polytechnique Fédérale de Lausanne; 4 Signal Processing Laboratory, Ecole Polytechnique Fédérale de Lausanne, Lausanne University Hospital

## Abstract

**Background:**

Widespread (Klauser et al., 2016) and progressive (Cropley et al., 2017) cerebral anomalies of white matter diffusion properties (i.e. fractional anisotropy, FA) have been observed in the Australian Schizophrenia Research Bank (ASRB), one of the largest samples of patients with schizophrenia. From a topological perspective, widespread alterations of white matter tend to concentrate into hub regions that interconnect brain areas over long-distances in a so-called “rich-club” (van den Heuvel et al., 2013; Klauser et al., 2016) in which the metabolic demand is high and thus are most likely to suffer from oxidative stress. Evidence from human and animal models suggests that redox dysregulation leading to oxidative stress during neurodevelopment is implicated in schizophrenia pathogenesis (Steullet et al., 2017). At the cellular level, the triad composed of NMDAR hypofunction, neuroinflammation and dopamine dysregulation interacts with redox imbalance and leads to oxidative stress, affecting oligodendrocytes precursor cells (OPC) and parvalbumine interneurons (Steullet et al., 2016). However, the links between redox imbalance, oligodendrocytes and gross alterations of white matter integrity are largely unexplored. Under oxidative stress induced in vitro by impairing the synthesis of glutathione (GSH), the key player in antioxidant defense, OPC showed a decreased proliferation mediated by an upregulation of Fyn kinase activity. In the prefrontal cortex of a mouse model with impaired GSH synthesis, mature oligodendrocyte numbers as well as myelin markers were decreased at peripuberty (Monin et al., 2014). FA was also reduced in fornix-fimbria and anterior commissure, a change accompanied by a reduced conduction velocity (Corcoba et al., 2015).

**Methods:**

49 patients with psychosis and 64 healthy controls were scanned with the same 3-Tesla scanner. The diffusion spectrum imaging (DSI) sequence included 128 diffusion-weighted images with a maximum b-value of 8000 s mm−2. White matter diffusion properties were estimated using generalized fractional anisotropy (gFA). Total blood cysteine (Cys, protein-bound form, free reduced and free oxidized form), the rate-limiting precursor of GSH, was measured by high performance liquid chromatography from plasma samples collected at the same time-point as MRI brain scans. Whole brain voxel-based analyses were performed using cluster-based non-parametric permutation testing on gFA maps. Cerebral levels of GSH were assessed by localized 1H-MRS measurements from a volume of interest in medial prefrontal cortex.

**Results:**

As previously described in ASRB, we observed widespread abnormalities of white matter in patients. Interestingly, the degree of white matter alterations (i.e. decreased gFA) patients could be predicted by the levels of blood cysteine, a precursor of GSH, strongly suggesting the important role played by oxidative stress in the pathophysiological mechanism. Also, we found that white matter alterations could be reversed by 6 months of add-on treatment with the antioxidant and GSH precursor N-acetyl-cysteine (NAC). Most importantly, this improvement was positively correlated with an increase in prefrontal GSH levels.

**Discussion:**

We propose that developmental redox imbalance inducing oxidative stress may lead to impairments of oligodendrocytes, myelin formation and eventually to the disruption of fibers integrity and conductivity, especially in brain regions having high metabolic demand. In patients, alterations of white matter are inversely correlated with blood levels of GSH precursor cysteine and could be prevented by the early administration of the antioxidant NAC.

